# Duodenal perforation due to a kink in a nasojejunal feeding tube in a patient with severe acute pancreatitis: a case report

**DOI:** 10.1186/1752-1947-4-162

**Published:** 2010-05-28

**Authors:** Zhihui Tong, Weiqin Li, Xinying Wang, Xianghong Ye, Ning Li, Jieshou Li

**Affiliations:** 1 Research Institute of General Surgery, Jinling Hospital, 305 East Zhongshan Road, Nanjing 210002, Jiangsu Province, China

## Abstract

**Introduction:**

Nasojejunal feeding tube placement can be achieved by fluoroscopic or endoscopic techniques. Significant complications due to nasojejunal feeding tube placement, such as hydrothorax, duodenal perforation and retroperitoneal emphysema, are very rare. We present a case of massive retroperitoneal emphysema and abscess because of duodenal perforation caused by a kink in a nasojejunal feeding tube.

**Case presentation:**

A 34-year-old Chinese woman was admitted to our intensive care unit due to hypertriglyceridemia and severe acute pancreatitis. As she suffered from acute respiratory distress syndrome and required mechanical ventilation, a nasojejunal feeding tube was placed by transnasal endoscopic technique. The procedure took place at her bedside. Half a month later, she had a high fever and abdominal distension. An abdominal radiography was performed and showed that the nasojejunal feeding tube was kinking on the third portion of the duodenum and the tip of the nasojejunal feeding tube was inserted into the right retroperitoneum on the second portion of the duodenum.

**Conclusion:**

When a nasojejunal feeding tube is placed through the transnasal endoscopic technique, an abdominal radiography should be used to confirm the tube's position and indicate if it is kinking or beyond the ligament of Treitz.

## Introduction

Enteral nutrition (EN) via a nasojejunal feeding tube (NJT) is a rational and acceptable method of nutritional support in patients with severe acute pancreatitis (SAP) [1-3]. NJT placement can be achieved through fluoroscopic or endoscopic techniques [4]. Importantly, in patients with SAP, the NJT should be beyond the ligament of Treitz [5] and the NJT should not be kinked. Significant complications due to NJT, such as hydrothorax, duodenal perforation and retroperitoneal emphysema, are very rare [6,7]. We report a case of massive retroperitoneal emphysema and an abscess caused by a duodenal perforation that resulted from a kink in a NJT.

## Case presentation

**Figure 1:**
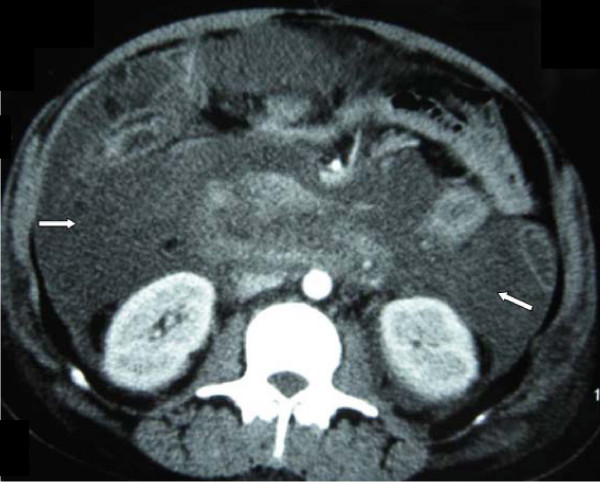
**Contrast-enhanced computed tomography of pancreas when the patient was admitted**. Enlarged necrotic pancreas and peripancreatic inflammation and fluid were shown in this picture. (arrows).

**Figure 2:**
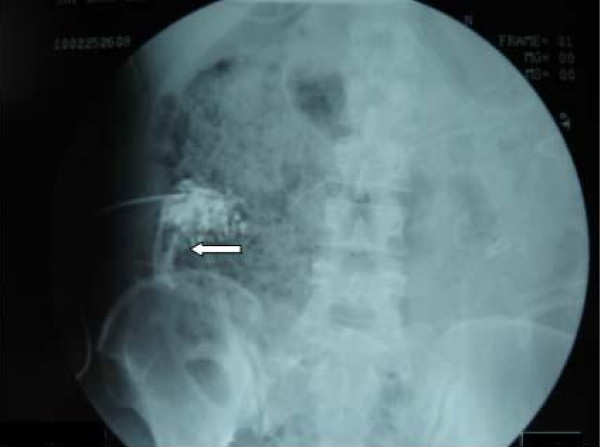
**Abdominal radiography of this patient**. Meglumine Diatrizoate was injected via the right drainage catheter (left arrow). The duodenum did not show up after injection.

**Figure 3:**
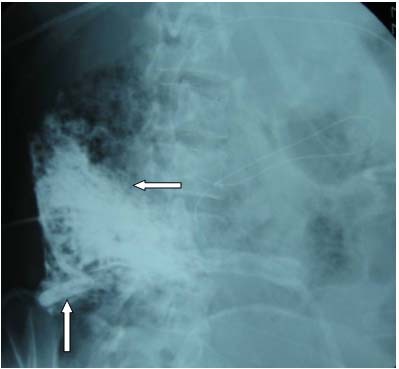
**Abdominal radiography of this patient**. Meglumine Diatrizoate was injected via NJT. The tip of the NJT was inserted into the right retroperitoneal abscess on the second portion of the duodenum (left arrow), and the right drainage catheter showed up (up arrow).

A 34-year-old female Chinese patient was admitted to our intensive care unit due to hypertriglyceridemia and severe acute pancreatitis. A contrast-enhanced computed tomography (CECT) on admission showed an enlarged necrotic pancreas and peripancreatic inflammation and fluid (Figure 1). As she suffered from acute respiratory distress syndrome (ARDS) and required mechanical ventilation, a NJT was placed on the fifth day after admission at bedside by transnasal endoscopic technique. The tube position was not determined by an abdominal radiography. We initiated a D5W solution feeding after intubation. No initial discomfort was noted as the peptisorb liquid (of up to 50 ml per hour) was administered. The patient tolerated this for 15 days, and was weaned from mechanical ventilation on the 10th day. However, on the 21st day after hospitalization, she again had a high fever and abdominal distension. So we checked her using an ultrasound (US) at bedside and performed a US-guided percutaneous catheter drainage of the doubtful retroperitoneal abscesses. The left abscess was demonstrated by the aspirate, but the aspirate from the right retroperitoneal abscess was similar to the peptisorb liquid. We injected 20 ml of methylthioninium chloride through the NJT and found it effusing immediately via the right drainage catheter. At first we thought that the right drainage catheter had penetrated the duodenum or superior segment jejunum. An abdominal radiography was performed and showed that the NJT was kinking on the third portion of the duodenum. The duodenum was not visualized when meglumine diatrizoate was injected via the right drainage catheter (Figure 2). Meglumine diatrizoate was injected via the NJT and showed that the tip of the NJT was inserted into the right retroperitoneal abscess on the second portion of the duodenum (Figure 3). This demonstrated that the duodenal perforation was caused by the NJT. The position of the NJT was adjusted using fluoroscopic guidance and the tip was exactly beyond the ligament of Treitz. After this, the peptisorb liquid from the right drainage catheter disappeared. But her symptoms did not release after two days of conservative treatment. A CT scan of the abdomen showed retroperitoneal emphysema and an abscess. An emergency exploratory laparotomy was performed. The duodenum perforation was not found but peptisorb liquid accumulation was identified. The retroperitoneal abscess was drained. The recovery course was smooth and EN via the NJT was started five days after the surgical procedure.

## Discussion

In patients with SAP, EN via NJT beyond the ligament of Treitz has been shown to result in significantly fewer septic events and fewer overall complications [1,2]. An NJT was placed on the fifth day after admission in order to provide EN to this patient. However, she suffered from ARDS and could not be transported to the radiology department. Thus, the NJT was placed at bedside using the transnasal endoscopic technique. However, abdominal radiography could not be used to demonstrate the position of the tube, whether it was kinking or beyond the ligament of Treitz.

Endoscopic NJT placement is a simple and effective method. We have experienced over a 96% success rate using this technique [4]. However, pushing the NJT blindly beyond the second portion of the duodenum may result in a kink in the distal end, especially in patients with stenosis of the duodenum or swelling in the tail of the pancreas. Endoscopic related pneumoretroperitoneum due to duodenal perforation has been reported [7]. But in this patient, we initially thought that the duodenal perforation was not a complication of endoscopic NJT placement because it was not until the 16th day after placement that she had a high fever and abdominal distension. The patient tolerated EN well for 15 days. The duodenum is the lack of serous membrane. When it is surrounded by peripancreatic inflammation and fluid, it becomes swollen and fragile. In this patient, the NJT was kinking on the third portion of the duodenum. Additionally, the tip of the NJT had collided with the wall of the duodenum on the second portion for a long time. Duodenal perforation may occur when the pressure of the tip is not removed in time. Once the tip of the NJT was inserted into the retroperitoneum and EN was not stopped, retroperitoneal emphysema and an abscess occurred immediately. When NJT is placed using endoscopic techniques, the final position must be determined by an abdominal radiography and interpreted by the attending radiologist.

The treatment for duodenal perforation is simple. The duodenum perforation may be cured spontaneously once the NJT is removed from the perforation, and the abscess is drained thoroughly. This is what happened with our patient.

## Conclusion

When an NJT is placed at bedside by a transnasal endoscopic technique, the tube position should be demonstrated by abdominal radiography to determine whether there is kinking or if it is beyond the ligament of Treitz.

## Consent

Written informed consent was obtained from the patient for publication of this case report and accompanying images. A copy of the written consent is available for review by the journal's Editor-in-Chief.

## Competing interests

The authors declare that they have no competing interests.

## Authors' contributions

ZT wrote the article, participated in the sequence alignment and drafted the manuscript. WL participated in the sequence alignment, formatted the pictures and performed language corrections. XW and XY collected the data and investigation studies, participated in the article design and critically evaluated the article. NL and JL conceived the study, and participated in its design and coordination and helped to draft the manuscript. All authors read and approved the final manuscript.
